# Comparative Analysis of the Bacterial Membrane Disruption Effect of Two Natural Plant Antimicrobial Peptides

**DOI:** 10.3389/fmicb.2017.00051

**Published:** 2017-01-23

**Authors:** Attila Farkas, Gergely Maróti, Attila Kereszt, Éva Kondorosi

**Affiliations:** Institute of Biochemistry, Biological Research Center of the Hungarian Academy of SciencesSzeged, Hungary

**Keywords:** nodule-specific cysteine-rich plant peptides (NCRs), antimicrobial effect, membrane disruption, foodborne pathogens, peptide localization

## Abstract

In the *Medicago truncatula* genome about 700 genes code for nodule-specific cysteine-rich (NCR) small peptides that are expressed in the symbiotic organ, the root nodule, where they control terminal differentiation of the endosymbiotic rhizobium bacteria to nitrogen-fixing bacteroids. Cationic NCR peptides were predicted to have antimicrobial activities. Here antibacterial activities of NCR247, NCR335, polymyxin B (PMB), and streptomycin were investigated and compared on two foodborne pathogens *Salmonella enterica* and *Listeria monocytogenes* as representatives of Gram-negative and Gram-positive bacteria. The integrity of the bacterial membrane was seriously compromised by these NCR peptides. Different localization was observed for NCR247 and NCR335 in the treated bacteria, the peptides mostly accumulated in the cytosol in *S. enterica* while they remained in the bacterial membrane in *L. monocytogenes*. Scanning electron microscopy revealed distinct membrane morphology of the peptide-treated bacteria. Complete cell disruption was induced by PMB and NCR335 in *S. enterica* while NCR247 treatment resulted in extensive budding observed on the cell surface of *Salmonella*. PMB had no effect on *L. monocytogenes* while NCR335 and NCR247 provoked morphological changes on this bacterium, the whole *Listeria* cell content was released in response to NCR335 treatment.

## Introduction

Antimicrobial peptides (AMPs) are considered as natural antibiotics produced by all kinds of living organisms including bacteria (Joerger, 2003; Hassan et al., 2012), plants (Benko-Iseppon et al., 2010), and animals (Hancock and Scott, 2000). Hundreds of AMP molecules have been isolated from prokaryotes to humans and plants (Maróti et al., 2011). They represent a cornerstone of the innate immune system in higher eukaryotes. Different AMPs show variations in their antimicrobial activity spectrum which ranges from Gram-positive and Gram-negative bacteria (including multidrug resistant pathogens) to viruses and fungi. AMPs are in use for hundreds of millions of years in nature and yet they have remained effective (Zasloff, 2002) suggesting that development of resistance against AMPs is moderate. However, experimental evolution has demonstrated the capability of bacterial populations to evolve resistance to AMP drugs (Perron et al., 2006). Insights into AMPs natural environment and their possible natural roles have shown that these peptides protect the host against pathogenic microorganisms and are also responsible for fine-balancing interactions with commensal and symbiotic bacterial populations and thus they enhance bio-diversity in microbial communities (Maróti et al., 2011).

The antimicrobial activities and mechanisms of AMPs are diverse. Cationic AMPs can interact with electronegative bacterial cell surface resulting either in cell lysis or disruption of bacterial membrane leading to transient pore formation and peptide transport inside the cell (Epand and Vogel, 1999; Jenssen et al., 2006). AMPs can have multiple intracellular targets when entering the cells. They can associate with nucleic acids, proteins or lipids and inhibit DNA, RNA, protein or cell wall synthesis (Ganz, 2003; Brogden, 2005; Hancock and Sahl, 2006; Hale and Hancock, 2007). Particular AMP targets include DNA gyrase, the heat-shock protein DnaK, the peptidoglycan precursor lipid II and even ATPase (Brogden, 2005; Hasper et al., 2006; Hilpert et al., 2010; Sass et al., 2010). AMPs were also reported to interfere with the cell cycle of fungal cells (Lobo et al., 2007).

Plants represent an extremely abundant and largely unexplored source of AMPs. The recent development of powerful high-throughput genomic techniques has disclosed the presence of large numbers, often even several hundreds of AMP-like genes in various plant genomes (Manners, 2007) indicating the importance of AMPs in the eukaryotes and particularly in plants. The innate immune system is a generic defense response of plants to keep off invading microbes and AMPs are essential part of it. AMPs can be expressed constitutively in specific plant organs or induced by microbes both at the site of infection and systemically (Sels et al., 2008). Interestingly, most of the AMPs are cysteine-rich including defensins, thionins, and cyclotides (Silverstein et al., 2007).

Symbiosis is a ubiquitous phenomenon on Earth, microbial symbionts inhabit various parts of most multicellular species and engage in both beneficial and harmful partnerships. Rhizobium-legume symbioses represent excellent models to study the transitions between mutualistic, parasitic, and free-living lifestyles. Legumes are unique in their ability to establish symbiotic interactions with rhizobium bacteria and to develop a new organ, the nitrogen-fixing root nodule. In certain species, inside the plant cells the bacteria are irreversibly converted to polyploid, non-dividing nitrogen-fixing bacteroids with altered properties of the cell envelope (Mergaert et al., 2006; Kondorosi et al., 2013). Terminal differentiation of bacteroids is controlled by symbiosis specific plant peptides in inverted repeat-lacking clade (IRLC) legumes (Mergaert et al., 2003; Alunni et al., 2007; Nallu et al., 2014). In the *Medicago truncatula* genome close to 700 genes code for nodule-specific cysteine-rich (NCR) peptides. The NCR peptides are composed of a relatively conserved signal peptide and a highly variable 20–50 amino acid long secreted peptide with four or six conserved cysteine residues. Due to the difference in amino acid composition, NCR peptides can be cationic, neutral, or anionic. NCRs resemble in their structure to defensins, the major class of plant AMPs and even certain cationic NCR peptides possess strong antimicrobial activity *in vitro* (Van de Velde et al., 2010; Tiricz et al., 2013; Ördögh et al., 2014). The NCR genes are exclusively expressed in the rhizobium-infected nodule cells, however, different sets of NCRs are induced during the progressive differentiation program of the symbiotic cells (Roux et al., 2014). It was demonstrated that the synthetic cationic NCR247 and NCR335 peptides provoke fast killing of various bacteria and fungi and thereby they are efficient natural antimicrobials (Tiricz et al., 2013; Farkas et al., 2014; Ördögh et al., 2014). As cationic peptides can interact with the negatively charged bacterial membranes our study focused on the membrane disruptive effect of NCR247 and NCR335 on two pathogenic bacterium strains, the Gram-negative *Salmonella enterica* and the Gram-positive *Listeria monocytogenes*. The effects of these symbiotic peptides were compared to those of two classical antibiotics, the prokaryotic translation inhibitor streptomycin (STM) and polymyxin B (PMB), a cyclic peptide antibiotic.

## Materials and Methods

### *In silico* Peptide Analysis

Two different antimicrobial peptide predictor tools were used; the Antimicrobial Peptide Database with APD3 algorithm: Antimicrobial Peptide Calculator and Predictor http://aps.unmc.edu/AP/ (Wang et al., 2016) and the AMP predictor tool of the Collection of Anti-Microbial Peptides (CAMP) (Thomas et al., 2009). The latter operates with four different prediction models taking into account the sequence composition, physico-chemical properties, and structural characteristics of amino acids; Support Vector Machine (SVM), Random Forest (RF), Artificial Neural Network (ANN), and Discriminant Analysis (DA) (Waghu et al., 2014). As a result, SVM, RF, and DA models give a probability score (between 0 and 1) (Waghu et al., 2016). Higher score means greater possibility for the peptide to exert antimicrobial activity. AMP: the sequence predicted to be antimicrobial. NAMP: the sequence predicted to be not antimicrobial.

### Microbial Strains and NCR Peptides

*Salmonella enterica* serovar Enteritidis (ATCC 13076) and *L. monocytogenes* (ATCC 19111) were purchased from validated culture collections. NCR247 and NCR335 were synthesized by conventional solid phase peptide synthesis at >95% purity, synthesis was done by ProteoGenix SAS (France), vendor provided data of peptide characterization including HPLC and Mass Spectrometry data.

### Determination of the Minimal Inhibitory Concentration (MIC) and Minimal Bactericidal Concentration (MBC) Using Broth Resazurin Microdilution Assay

A single colony from both strains were grown in Mueller Hinton Broth (MHB) at 37°C overnight. The starter cultures were diluted and grown until OD_600_ = 0.5–1.0. The number of colony-forming units was determined, the dilution factors necessary for performing the MIC tests were calculated and the dilutions were made to obtain cultures with 5 × 10^6^ cfu/mL. Plates were prepared under aseptic conditions. A sterile 96-well plate was labeled. A volume of 90 μL of sterile MHB was pipetted into each well of first row of the plate, these wells served as negative controls. Fifty microliters MHB was added to all other wells. Twofold dilution series of the NCR247, NCR335, PMB, and STM were prepared (0.1, 0.5, 1, 2, 4, 8, 16, 32, 64, and 128 μM) and added to the wells. Ten microliters of resazurin indicator solution (0.1% diluted in MHB) was added into each well. Finally, 10 μL of bacterial suspension (5 × 10^6^ cfu/mL) was added to each well to achieve a concentration of 5 × 10^5^ cfu/mL. The plates were prepared in triplicate and incubated at 37°C for 20 h. The color change was then assessed visually. Any color changes from purple to pink or colorless were recorded. The lowest peptide/PMB/STM concentration at which color change occurred was considered as the MIC value (Wiegand et al., 2008; Balouiri et al., 2016). For the determination of MBC, 100 μL cultures were plated from the wells that exhibited no growth. The plates were incubated at 37°C for 24 h. The lowest concentration where no bacterial growth was detected was considered as MBC (Kang et al., 2011).

### Determination of the Complete Elimination Concentrations (CE Values) Using Drop Plate Method

Both strains were grown in 10 mL of liquid Luria Broth (LB) at 37°C overnight, then the starter cultures were diluted in 10 mL LB to OD_600_ = 0.05 and grown until mid-logarithmic phase (OD_600_ = 0.5–0.8). The optical density was set to OD_600_ = 0.1 in 20 mM potassium phosphate buffer, pH 7.2 (PB) for the antimicrobial tests. For *S. enterica*, the OD_600_ of 0.1 represents 8.25 × 10^7^ CFU/mL, for *L. monocytogenes* 3.07 × 10^9^. The antimicrobial activities were determined according to the guidelines of the drop plate method (Herigstad et al., 2001; Chen et al., 2003). Peptides were serially diluted in PB buffer. Fifty microliter (μL) of bacterial culture was mixed with 50 μL NCR peptide/STM/PMB applied at 0.5, 2.5, 5, 10, 25, and 50 μM final concentrations followed by incubation at room temperature for 2 h without shaking. Treated bacteria were dropped on LB agar plates. The drops were absorbed to agar in less than a half an hour and the plates were incubated at inverted position at 37°C overnight. Three independent experiments were performed in all cases.

### Membrane Permeability Assay

Cytoplasmic membrane damage was assayed with the Live/Dead BacLight bacterial viability kit (Invitrogen L7012) according to the manufacturer’s instructions (Horváth et al., 2015). Bacteria (*S. enterica* and *L. monocytogenes)* were grown to mid-logarithmic phase and the optical density was set to OD_600_ = 0.1 in LB and washed gently with PB buffer. Bacterial cells resuspended in PB buffer were treated with 10 μM NCR247, 5 μM NCR335, and 5 μM PMB at room temperature for 60 min. Untreated cells served as negative control. Then the cells were stained with 7.5 μM SYTO-9 and 30 μM propidium iodide (PI). After 15 min incubation in dark the cells (5 μL) were spotted on microscope slide and covered with 2% (w/v) agar slices and observed with Olympus Fluoview FV 1000 confocal laser microscope with 60× magnification objective. 488 nm laser was used for excitation, and emission was detected at 500–530 nm for SYTO9. Excitation and emission wavelengths were 543 and 555–655 nm for PI, respectively. Sequential scanning was used to avoid crosstalk of the fluorescent dyes.

### Localization of FITC-Labeled Peptides in Bacteria

Mid-logarithmic phase bacterial cultures OD_600_ = 0.1 were resuspended in PB buffer containing FITC-labeled NCR247 or NCR335 at a concentration corresponding to 0.5 × CE. Following incubation for 30 min at room temperature, samples were co-stained with 800 nM FM4-64 membrane dye. The stained cells (5 μL) were spotted on a microscope slide and covered with 2% (w/v) agar slices. Localization of the labeled peptides was observed with Olympus Fluoview FV 1000 confocal laser microscope with 60× magnification objective (Farkas et al., 2014). 488 nm laser was used for excitation, and the emission was detected at 500–530 nm for FITC. Excitation and emission wavelengths for FM4-64 were 543 and 555–655 nm, respectively. Sequential scanning was used to avoid crosstalk of the fluorescent dyes.

### Scanning Electron Microscopy (SEM)

Mid-logarithmic phase bacteria were washed twice with and resuspended in PB buffer at OD_600_ = 0.1. The cells were incubated at room temperature with 0.5 × CE NCR247 and NCR335 for 5 min and 2 h. Cells were then fixed with 2.5% (v/v) glutaraldehyde and 0.05 M cacodylate buffer pH 7.2 in phosphate buffered saline (PBS), and post fixed with 0.1% osmium tetroxide in PBS for 1 h. Five microliters of the above bacterial suspension were spotted on a polycarbonate membrane filter (GTTP 0.2 μm, Millipore). Thereafter, the filters were washed twice with PBS and dehydrated with a graded ethanol series (30%, 50%, 70%, 80%, and 100% ethanol, each for 15 min). Untreated bacterial cells handled in the same way served as control. The samples were dried with a critical point dryer, followed by 12 nm gold coating and observed under a scanning electron microscope (JEOL JSM-7100F/LV).

## Results

### *In silico* AMP Characteristics of Symbiotic NCR247 and NCR335

Previous works demonstrated antimicrobial activity of NCR247 and NCR335. Their classification as AMPs required in-depth analysis of their sequence-based physico-chemical properties, such as peptide length, molecular weight, amino acid composition, hydrophobicity, charge, and Boman index (**Table 1**). The length parameter influences the insertion ability of peptides into target membranes (Shai, 2002). Both NCR peptides are short, NCR247 is composed of 24 aa, while the active form of NCR335 is 64 aa. Cysteines contribute to the biochemical stability of the molecules by forming disulfide bridges. Both NCR247 and NCR335 have four cysteine residues which form two inter-chain disulfide bridges. High positive net charge provokes interactions with the negatively charged microbial membranes which is another important factor to exert antimicrobial activity (Tossi et al., 2000). NCR335 has one of the highest positive net charge (+14) among all *M. truncatula* NCRs, while NCR247 has a net charge of +6. The cationic amino acids lysine (K) and arginine (R) flanked by hydrophobic, membrane-insertive isoleucine (I), valine (V), leucine (L), phenylalanine (F), and alanine (A) residues form amphipathic structure with 29% and 37% hydrophobic ratio in NCR247 and NCR335, respectively. The Boman index, which is a hydropathy numeric value, measures peptides affinity to other proteins. The Boman index of NCR247 is 4.63 kcal/mol, which is the highest value among all NCRs (Farkas et al., 2014), while that of NCR335 is 2.88 kcal/mol. The assessment of the physico-chemical properties of NCR247 and NCR335 revealed clear AMP properties. Additionally, all tested AMP prediction tools at the CAMP, such as RF, SVM, ANN, and DA (Thomas et al., 2009) classified NCR247 as AMP with high probability, while NCR335 was recognized as AMP by two of the four models (**Table 2**).

**Table 1:** Physico-chemical properties of NCR247 and NCR335.

NCR247 sequence	RNGCIVDPRCPYQQCRRPLYCRRR	

**NCR335 sequence**	**RLNTTFRPLNFKMLRFWGQNRNIMKHRGQKVHFSLILSDCKTNKDCPKLRRANVRCRKSYCVPI**	
	
	**NCR247**	**NCR335**
Amino acid	24	64
Molecular weight	2991.574 Da	7718.24 Da
Amino acid composition	Hydrophobic amino acid – I: 1, V: 1, L: 1, F: 0, C: 4, M: 0, A: 0, W: 0, The number of G and P – G: 1, P: 3, Negatively charged amino acid – E: 0, D: 1, Positively charged amino acid – K: 0, R: 7, H: 0, Other amino acid – T: 0, S: 0, Y: 2, Q: 2, N: 1	Hydrophobic amino acid – I: 3, V: 3, L: 6, F: 4, C: 4, M: 2, A: 1, W: 1, The number of G and P – G: 2, P: 3, Negatively charged amino acid – E: 0, D: 2, Positively charged amino acid – K: 7, R: 9, H: 2, Other amino acid – T: 3, S: 3, Y: 1, Q: 2, N: 6
Hydrophobic ratio	29%	37%
Net charge	+6	+14
Protein-binding Potential (Boman index)	4.63 kcal/mol	2.88 kcal/mol


*The parameters were calculated using the antimicrobial peptide database with APD3 algorithm: Antimicrobial Peptide Calculator and Predictor (http://aps.unmc.edu/AP).*

**Table 2:** Antimicrobial peptide prediction of NCR247 and NCR335 peptides.

	Seq. ID.	Class	AMP probability
Support Vector Machine (SVM) classifier	NCR247	AMP	0.970
	NCR335	AMP	0.858
Random Forest Classifier	NCR247	AMP	0.7275
	NCR335	NAMP	0.439
Artificial Neural Network (ANN) classifier	NCR247	AMP	
	NCR335	AMP	
Discriminant Analysis classifier	NCR247	AMP	0.997
	NCR335	NAMP	0.405


*All four tested models of the Collection of Anti-Microbial Peptides (CAMP) predictor tool classified NCR247 as AMP with high probability, while NCR335 was recognized as AMP by two of the four models. NAMP refers to non-AMP.*

### Differential Sensitivity of Bacteria to NCRs and Classical Antibiotics

**FIGURE 1:**
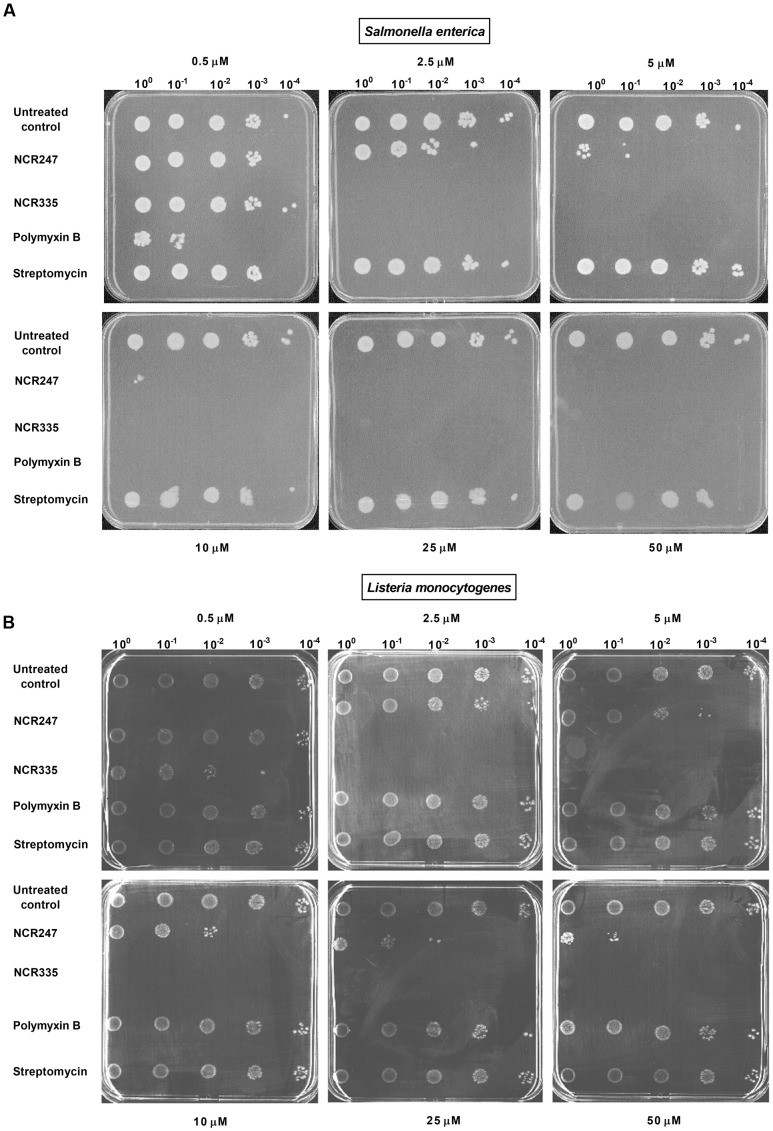
**Antimicrobial activity of nodule-specific cysteine-rich (NCR) peptides.** **(A)** Activity against *Salmonella enterica*. **(B)** Activity against *Listeria monocytogenes*. Complete elimination (CE) values of 2.5 μM were determined for NCR335 against both *S. enterica* and *L. monocytogenes*. The CE values of NCR247 were ≥10 μM against *S. enterica*, while *L. monocytogenes* was less sensitive to this peptide (CE > 50 μM). *L. monocytogenes* was fully resistant to both polymyxin B and streptomycin while showed clear sensitivity to the plant peptides.

Antimicrobial activity of NCR247 and NCR335 peptides against *S. enterica* and *L. monocytogenes* has been investigated and compared to that of two well-characterized antimicrobials, PMB and streptomycin (STM). Two different approaches were applied to characterize the peptides antimicrobial features (**Figure 1** and **Table 3**). The standard MIC, MBC assay using microtiter plates with various concentrations of peptides and antibiotics was used to analyze bacterial growth inhibition. The MIC value obtained for PMB was 0.5 μM on *S. enterica* and 128 μM for *L. monocytogenes* (**Table 3**). The MBC values for PMB was 1 μM on *S. enterica* and higher than 128 μM for *L. monocytogenes*. STM inhibited *Salmonella* growth at 4 μM (MIC) and eliminated all *Salmonella* bacteria at a threefold greater concentration of 32 μM (MBC). STM was not able to inhibit the growth of *L. monocytogenes* up to 128 μM. The MIC values of NCR247 and NCR335 were 32 and 16 μM against *S. enterica*, respectively. The MBC values for NCR247 and NCR335 on *S. enterica* were 64 and 16 μM, respectively. The MIC values of NCR247 and NCR335 were 128 and 32 μM against *L. monocytogenes*, respectively. The MBC value of NCR247 on *L. monocytogenes* was higher than 128 μM, while NCR335 had an MBC of 32 μM on this Gram-positive pathogen (**Table 3**).

**Table 3:** Minimal inhibitory concentration (MIC) and minimal bactericidal concentration (MBC) of NCR247 and NCR335 on *Salmonella enterica* and *Listeria monocytogenes* using broth resazurin microdilution assay.

MIC and MBC (μM)				

**Peptide/Antibiotics**	**Gram-negative**		**Gram-positive**	
		
	***S. enterica***		***L. monocytogenes***	
		
	**MIC**	**MBC**	**MIC**	**MBC**
NCR247 (μM)	32	64	128	>128
NCR335 (μM)	16	16	32	32
Polymyxin B (μM)	0.5	1	128	>128
Streptomycin (μM)	4	32	>128	NE


*MIC and MBC values were determined for the NCR peptides and for PMB and STM using a standard, validated method. NE: no effect up to 128 μM.*

Using the drop-plate approach *S. enterica* was resistant to STM but was efficiently killed by PMB and NCR335. PMB reduced colony formation already at 0.5 μM and at 2.5 μM both PMB and NCR335 eliminated all bacteria. NCR247 reduced bacterial growth at 2.5 μM, but complete elimination (CE) required a peptide concentration of at least 10 μM (**Figure 1A**). Interestingly, *L. monocytogenes* was fully resistant to both PMB and streptomycin while showing clear sensitivity to the plant peptides. NCR335 already at 2.5 μM killed all bacteria, while NCR247 reduced growth from 10 μM, nevertheless, even at 50 μM the inhibition was not complete (**Figure 1B**). Based on these data, the CE concentrations for *S. enterica* were ≤2.5 μM for NCR335 and PMB, while ≥10 μM for NCR247. CE for *L. monocytogenes* was ≤2.5 μM in the case of NCR335, while CE of NCR247 was more than 50 μM (**Table 4**).

**Table 4:** Complete elimination (CE) concentrations of NCR247 and NCR335 on *S. enterica* and *L. monocytogenes* using drop plate method.

Drop plate method		

**Peptide/antibiotics**	**Concentration of CE**	
	
	**Gram-negative**	**Gram-positive**
	
	***Salmonella enterica***	***Listeria monocytogenes***
NCR247 (μM)	≥10	>50
NCR335 (μM)	≤2.5	≤2.5
Polymyxin B (μM)	≤2.5	NE
Streptomycin (μM)	NE	NE


*NCR247 was active above 10 μM against *S. enterica* and above 50 μM against *L. monocytogenes*. NCR335 killed all the cells on the tested strains at 2.5 μM. Polymyxin B eliminated all *S. enterica* cells at a concentration less than 2.5 μM. *L. monocytogenes* was fully resistant to polymyxin B. Streptomycin had no effect on the tested bacteria. NE: no effect up to 50 μM.*

### NCR247 and NCR335 Increase Cell Membrane Permeability

**FIGURE 2:**
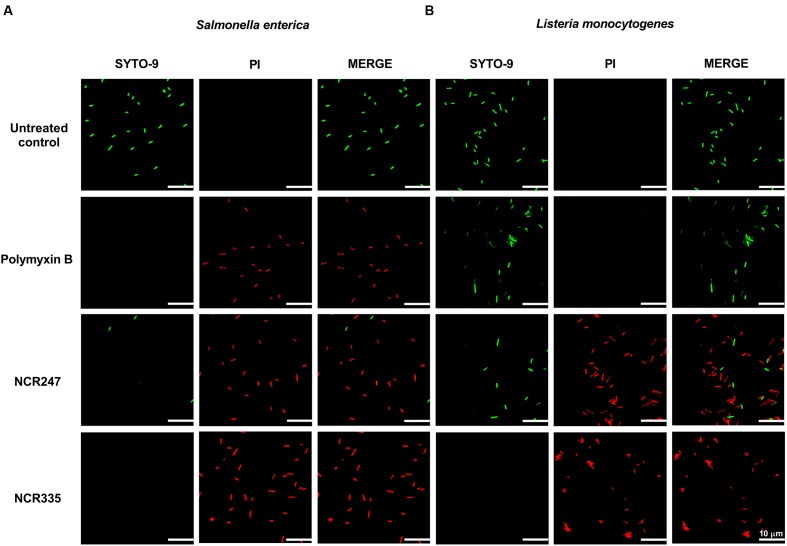
**Bacterial membrane integrity is compromised in the presence of NCRs.** Fluorescence microscopy images of **(A)** *S. enterica* cells and **(B)** *L. monocytogenes* cells treated with 10 μM NCR247, 5 μM NCR335, and 10 μM polymyxin B for 1 h and stained with SYTO-9 and PI dyes. Green fluorescence indicates unaltered membrane integrity. Red fluorescence indicates membrane disintegration (permeable membrane). Scale bar: 10 μm.

The damage of the bacterial membrane was visualized by co-staining of the cells with two fluorescent nucleic acid dyes. The membrane-permeable SYTO-9 stains all bacterial cells and shows green fluorescence. In contrast, propidium iodide (PI) enters only damaged non-living cells and produces red fluorescence. Bacterial cultures of *S. enterica* and *L. monocytogenes* were incubated for 60 min with 10 μM PMB, 10 μM NCR247, or 5 μM NCR335 (0.5 × CE values), after co-staining with SYTO9 and PI the cells were observed with confocal laser scanning microscopy (CLSM) (**Figure 2**). The untreated *S. enterica* and *L. monocytogenes* cells showed only green fluorescence indicating that all cells were alive. In contrast, the majority of *S. enterica* cells treated with PMB, NCR247 or NCR335 showed red fluorescence which indicated membrane damage. A significant degree of membrane permeabilization was induced on *L. monocytogenes* upon the addition of NCR247, while PMB was not able to induce membrane permeabilization on this Gram-positive pathogen. NCR335 provoked, similarly, red fluorescence as a consequence of membrane damage and death of *L. monocytogenes*. The assay clearly showed an increased permeability of both Gram-negative and Gram-positive membranes in response to treatment with NCR247 or NCR335. Furthermore, the results indicated that the bactericidal effect of NCR peptides on *S. enterica* and *L. monocytogenes* is directly realized through membrane permeabilization and damage.

### Localization of FITC-Labeled NCR247 and NCR335

**FIGURE 3:**
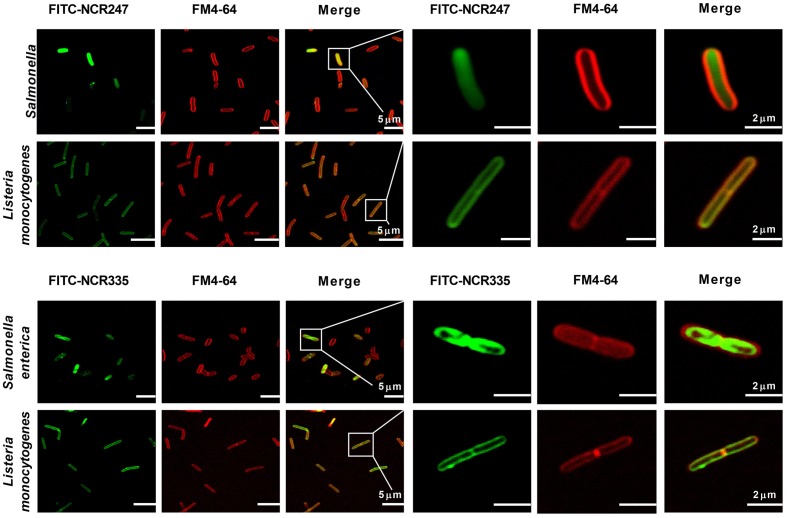
**Localization of FITC-NCR peptides.** Fluorescence confocal microscopy images of bacteria treated with FITC-labeled NCR peptides and stained with FM4-64 dye. Green fluorescence indicates the localization of the peptides in the cells. Red fluorescence shows the cytoplasmic membrane stained with FM4-64. Scale bar: 5 and 2 μm.

In addition to the membrane disrupting effects of AMPs at ≥CE, the peptides at lower concentration without membrane disruption can have various intracellular targets as it was shown for NCR247 (Farkas et al., 2014). To test if NCR247 and NCR335 interact only with the bacterial membrane or enter the cytosol, *S. enterica* and *L. monocytogenes* cells were treated with FITC-labeled NCR247 and NCR335 at 0.5 × CE for 30 min and co-stained with FM4-64 marking the bacterial membranes with red color (**Figure 3**). Both FITC-NCR247 and FITC-NCR335 showed slow penetration through the Gram-negative *S. enterica* membranes and accumulated in the bacterial cytoplasm. In contrast, the fluorescence of FITC-NCR247 and NCR335 and FM4-64 labels overlapped in *L. monocytogenes* indicating the membrane localization of NCR247 and NCR335 in this Gram-positive pathogen. Overall, the localization results suggested different membrane disruption mechanisms of NCR247 and NCR335 on Gram-positive and Gram-negative bacteria.

### NCR247 and NCR335 Causes Distinct Morphological Changes in *S. enterica* and *L. monocytogenes*

**FIGURE 4:**
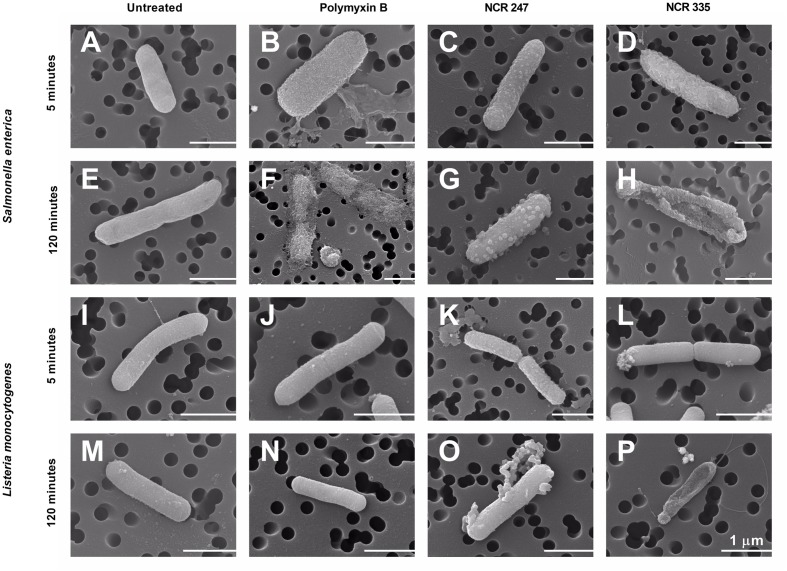
**Scanning electron microscopy investigations of peptide-treated *S. enterica* and *L. monocytogenes.*** Untreated bacterial membranes were intact **(A,E,I,M)**. Blisters were observed on the cell surface of *S. enterica* treated with NCR247 for 5 min **(C)**, while bleb-like structures appeared on the *Salmonella* cells after 2 h NCR247 treatment **(G)**. NCR335 and polymyxin B treatments for 5 min resulted in wrinkly surface on *S. enterica* **(B,D)**, while 2 h treatments with these peptides caused complete lysis of *S. enterica* cells **(F,H)**. *L. monocytogenes* was shown to be fully resistant to polymyxin B **(J,N)**, while *L. monocytogenes* cells treated with NCR247 and NCR335 showed rough surface after 5 min treatment **(K,L)**. Cell content was released and debris was visible around *L. monocytogenes* cells after 2 h treatment with NCR247 and NCR335 **(O,P)**. Scale bar: 1 μm.

Antimicrobial activity of AMPs is usually depending on their positive net charge and their capacity to bind to the negatively charged bacterial membranes. While NCR247 and NCR335 are both cationic their MIC, MBC, and CE values were significantly different suggesting that in addition to the positive charge their amino acid composition and primary sequence contribute also to their activities and might influence their interaction with the bacterial envelope. Therefore, we studied how the morphology of *S. enterica* and *L. monocytogenes* is affected by NCR247, NCR335, and PMB treatments at CE values (**Figure 4**). Interestingly, NCR247 and NCR335 displayed distinct localization as observed in **Figure 3**. Scanning electron microscopy (SEM) observation was applied to visualize the distinct morphological changes on the bacterial membranes treated with NCR247 and NCR335. The results showed clear differences in the membrane morphology of the untreated and peptide treated *S. enterica* and *L. monocytogenes*. The untreated bacterial membranes were smooth and intact (**Figures 4A,E,I,M**). *S. enterica* cells treated with 2.5 μM of PMB provoked leaking wrinkly cell surface within 5 min (**Figure 4B**) and complete cell lysis after 2 h (**Figure 4F**), while even a 10-fold higher concentration of PMB had no effect on *L. monocytogenes*. The cells remained intact and showed fairly normal surface either after 5 min (**Figure 4J**) and 2 h incubation (**Figure 4N**) with 25 μM PMB. However, clear changes were observed on *S. enterica* cells treated with 10 μM NCR247 for 5 min, the formation of blebs was observed throughout the *Salmonella* cell surface (**Figure 4C**). The effective NCR247 concentration for *L. monocytogenes* was higher than that for *S. enterica. L. monocytogenes* cells treated with 50 μM NCR247 showed leaking rough surface after 5 min treatment indicating the loss of membrane integrity (**Figure 4K**). Furthermore, cell debris accumulated from lysed *Listeria* cells was observable around the cells when 2 h treatment was applied (**Figure 4O**). NCR335 treatment for 5 min at 2.5 μM concentration resulted a wrinkled membrane surface on *S. enterica* (**Figure 4D**) similar to what was detected in the case of PMB treatment (**Figure 4B**). *L. monocytogenes* cells treated with NCR335 for 2 h at the same concentration (2.5 μM) provoked a complete loss of osmotic pressure and the cells lysed (**Figure 4P**). This drastic *Listeria* cell lysis in response to NCR335 treatment at 2.5 μM concentration was in good accordance with the results of the CE tests. The data suggested that both NCRs interacted with both Gram-negative and Gram-positive bacterial membranes and resulted in pore formation leading to cell death.

## Discussion

NCR247 and NCR335 peptides are members of the NCR peptide family discovered in *M. truncatula* (Mergaert et al., 2003; Nallu et al., 2014). In their natural environment, peptides concentrations of the symbiotic cells are incomparably lower and they alter the morphology and physiology of the endosymbiont bacteria without killing them. Based on physico-chemical AMP properties they primarily act via membrane disruption. However, identification of intracellular targets demonstrated that NCR247 efficiently inhibits bacterial cell division (Farkas et al., 2014). The detailed *in silico* characterization of these natural plant peptides suggested that NCR247 and NCR335 exerted antimicrobial features which was verified by multiple prediction algorithms and experimental studies. Previous work has shown that NCR247 and NCR335 induced membrane depolarization on cultured *S. meliloti* (Tiricz et al., 2013).

In this study, the potential antimicrobial activity and the bacterial membrane disruptive effects of NCR247 and NCR335 were tested on two pathogenic bacteria, the Gram-negative *S. enterica* and the Gram-positive *L. monocytogenes*. Salmonellosis (Löfström et al., 2016) and listeriosis (Lecuit, 2007) are among the most common foodborne diseases in the world, these foodborne pathogens infect human via transfer from animals and animal-derived food products and potentially cause serious diseases. Since these bacteria have the ability to develop resistance to multiple classes of antibiotics it is plausible that certain *Salmonella* and *Listeria* strains might evolve toward multiresistance. Novel antimicrobial agents effective against Gram-negative and/or Gram-positive pathogens are of high interest to develop efficient future treatments. AMPs represent a diverse group of synthetic and natural molecules with real potential to become effective future drugs as peptide antibiotics. A number of AMPs (mostly cationic ones) were shown to display remarkable antibacterial activities.

Antimicrobial peptides usually have a molecular mass less than 10 kDa and contain 2–8 positively charged amino acids, such as lysine and arginine that define their net positive charge at physiological pH. Furthermore, AMPs consist of up to 50% hydrophobic amino acids contributing to the amphipathic conformation when interacting with the target cell and binding to the membrane surface (Peters et al., 2010; Bahar and Ren, 2013). The outer membrane is the first interactor and the main target of the AMPs. Various modes of action have been described including membrane permeabilization through pore formation and membrane micellization in a detergent-like way (Shai, 1999). A number of studies reported that an increased membrane permeability alone might not be sufficient to cause cell death. AMPs also target intracellular compartments, such as various enzymes and ribosomes. Computational studies represent an integral part of AMP investigations. Several comparative analyses have demonstrated that AMPs have common motifs which correlate with specific parameters of their biological activity. These methods are based on peptide characteristics, such as primary sequence, charge, hydrophobicity, amphipathicity, size, and structure in order to obtain representative and relevant information on the relative contribution of each property to the peptide’s biological activity. Computational sequence analysis was used to determine the basic AMP characteristics of the legume peptides NCR247 and NCR335. Lysine and arginine positively charged amino acid residues are especially abundant in NCR247 and NCR335. Arginines are highly concentrated at the C-terminal part of NCR247. The high number of lysine and arginine residues is consistent with the cationic nature of AMPs (Brogden, 2005). Most cationic peptides have a positive net charge ranging from +2 to +9. Upon interaction with the membrane they bind to the lipid-peptide interface evolving strong electrostatic linkage with the negatively charged bacterial phospholipid membranes (Yount et al., 2006). NCR335 has one of the highest positive net charge (+14) among legume NCRs, while NCR247 has a net charge of +6. The majority of AMPs have a net charge ranging from +4 to +6, which represent an optimal charge for biological activity (Tossi et al., 2000; Giangaspero et al., 2001). Various studies pointed out that net charge and positively charged residues significantly affect the antimicrobial activity of α-helical AMPs (Tossi et al., 2000; Dathe et al., 2001; Giangaspero et al., 2001). Jiang et al. showed that decreasing the net charge on V13K analogs (less than +4) made the peptide inactive, while an increase in net charge from +4 to +8 resulted in higher antimicrobial activity and lower level hemolytic activity. However, further increase of net charge to +9 and +10 made the peptide more toxic (Jiang et al., 2008). Based on our present results and previous reports, it seems that NCR247 a good candidate for antimicrobial agent exerting antimicrobial activity without toxicity (Tiricz et al., 2013; Farkas et al., 2014). NCR335 has an especially high net charge explaining its strong antimicrobial activity. However, this peptide likely has a toxic effect on eukaryotic cells. Peptide hydrophobicity is also an important parameter for biological activity. Hydrophobicity is defined as the percentage of hydrophobic residues within a peptide, this value is around 50% for AMPs (Tossi et al., 2000; Yount et al., 2006). Two general requirements in terms of hydrophobicity have to be fulfilled by a membrane-lytic peptide: the peptide has to be soluble in water to enable rapid transport to the target microbes (low hydrophobicity required) and at the same time the peptide has to interact with the hydrophobic region of the bilayer in order to disturb the membrane structure and provoke permeability (high hydrophobicity required) (Dathe and Wieprecht, 1999). The total hydrophobic ratios of 29 and 37% for NCR247 and NCR335, respectively, is optimal for the peptides to attach to the membrane bilayer and to form hydrophobic moment to depolarize the membrane. Cysteine residues are abundant in the β-folded AMP family and these amino acids are important in terms of both structure and activity of these peptides. Cysteine amino acids are known to form disulfide bonds that confer stability to the peptide. Clear enrichment of cysteines was observed in both NCRs tested. The four cysteines form two disulfide bridges in NCR247 (Haag et al., 2011) and NCR335. The exact natural structure of NCRs disulphide bridges and their specific roles in the antimicrobial activity of the peptides are mostly unknown. It was shown for NCR247 that either cysteine replacements or the modifications of disulphide bonds altered the antimicrobial activity of the peptide at least against *Sinorhizobium meliloti* (Haag et al., 2012).

Bioinformatics prediction tools might be useful for large-scale screening and identification of novel potential AMPs. The Collection of Anti-Microbial (CAMP_R3_) database is an online server collecting information on sequence, protein definition, accession numbers, activity, source organism, target organisms, protein family. CAMP_R3_ currently has data on 10247 sequences, 757 structures, and 114 signatures present in 45 AMP families (Waghu et al., 2016). The database includes a prediction tool for AMP identification, this tool uses four models (SVM, RF, ANN, and DA). SVM, RF, and DA models each provides a probability score between 0 and 1 for the analyzed peptide sequence. Higher score represents greater possibility for the peptide to exert antimicrobial activity. In this study, the two natural NCR247 and NCR335 peptides clearly showed AMP characteristics and the prediction tool recognized NCR247 as true AMP by all four applied models, while NCR335 was predicted as AMP by two models (SVM and ANN).

Rapid killing of microbial targets is an important feature of AMPs. As reported here we determined the MIC, MBC, and CE values for two NCR peptides using a standard assay for MIC and MBC determination and another approach (drop plate method) for CE determination and further characterization of the antimicrobial features of the tested plant peptides. The killing effects of the two plant peptides were compared to those of two well described antimicrobials, the broad spectrum aminoglycoside antibiotic streptomycin (STM) and the peptide antibiotic PMB. STM is a protein synthesis inhibitor, it interrupts the ribosome cycle at the initiation of protein synthesis (Luzzatto et al., 1968). PMB alters bacterial outer membrane permeability by binding to a negatively charged site in the lipopolysaccharide layer (Velkov et al., 2010). After binding to lipopolysaccharide in the outer membrane of Gram-negative bacteria, polymyxins disrupt both the outer and inner membranes (Velkov et al., 2013). PMB is efficiently used against resistant Gram-negative infections as a last-line therapy while it has a weak or no effect on Gram-positive pathogens. Interestingly, the two methods resulted in different results, which implies to the importance of the applied conditions when determining antimicrobial efficiency. In general, the peptides showed lower antimicrobial efficiency with the standard MIC and MBC determination method compared to that obtained using the drop-plate method resulting in the CE values. The main reason for the observed difference is the composition of the medium where the antimicrobial action takes place. The standard method uses the rich growth medium MHB containing divalent cations in high concentrations, while the treatment takes place in a low-salt medium in the drop plate method. The inhibition by divalent cations is a general feature of cationic AMPs. As results of the drop-plate method both *S. enterica* and *L. monocytogenes* were resistant to STM, PMB had antimicrobial effect only on the Gram-negative *S. enterica*, while NCR247 and NCR335 exerted significant antimicrobial effect against both food-borne pathogens. The killing effect of NCR335 was more pronounced, than that of NCR247, which is probably related to the higher positive net charge of NCR335. The observed bacterial resistance to STM using the drop-plate method is interesting and indicates that STM needs significantly longer time to exert its effect compared to the effect of NCR peptides.

The cationic NCR247 and NCR335 were shown to have high affinity to bind to the bacterial membranes (either Gram-negative or Gram-positive). Interestingly, the peptides showed intracellular, cytoplasmic localization only the Gram-negative *S. enterica*, while the peptides remained in the membrane of the Gram-positive *L. monocytogenes* as revealed by fluorescent microscopy. Both cytoplasmic and intramembrane accumulations suggested that NCR247 and NCR335 depolarized the bacterial membranes, which was confirmed by a live/dead staining assay. Beside the membrane disruptive effect, NCR247 and NCR335 modulate the bacterial synthetic processes (Tiricz et al., 2013; Farkas et al., 2014). Both NCRs had clear antimicrobial effect on the Gram-positive *L. monocytogenes* as well, while PMB proved to be ineffective against this pathogen. Scanning electron microscopy has demonstrated the differential membrane effect of the two NCR peptides. Treatment of the Gram-negative *S. enterica* and the Gram-positive *L. monocytogenes* with NCR247 and NCR335, respectively, resulted in different membrane morphology. Blisters, bleb-like membrane surface changes, irregular-wrinkled membrane stacks, deep craters and bursts, lysed cells were clearly observed in the SEM micrographs. The results indicated different interactions of NCR247 and NCR335 with the *S. enterica* membrane. In response to NCR247 treatment at 0.5 × CE the *Salmonella* outer membrane was destabilized instantly and the peptide penetration resulted in blisters and later bleb-like structures as observed in the SEM micrographs. Firstly, the NCR247 peptide induced leakage of the inner membrane appearing as small blisters. As a next step complete disruption of the inner membrane and the release of the cell content into the periplasmic space were observed in response to NCR247 treatment. NCR335 treatment of *S. enterica* at 0.5 × CE resulted in cells with slightly wrinkled cell wall. *Salmonella* cells with rough surface were observed after 5 min treatment, while widespread cell lysis was detected after 2 h of treatment. The original rod-shaped morphology of *S. enterica* has been lost and deep bruises appeared on the cell surface. The wrinkled and rough surface and the deformation of the cell shape and cell wall can be associated with membrane permeabilization and the release of cell plasma. The effect of PMB on the *Salmonella* cell surface was similar to the effect of NCR335 suggesting similar mechanism of action. The Gram-positive *L. monocytogenes* was fully resistant to PMB, no signs of any antimicrobial effect could be observed by SEM as well. The SEM investigation revealed interesting differences between the untreated *Listeria* samples and the peptide treatments. While the untreated cells and the PMB treated cells displayed normal smooth cell surface, the cells treated by NCR peptides (either NCR247 or NCR335) showed a slightly rough surface with small blisters even after 5 min incubation. The effect of NCR335 was more pronounced than that of NCR247, which corroborated with the antibacterial effects of these peptides. Bulges of intracellular cell debris were observed after 5 min NCR335 treatment at 0.5 × CE and total cell lysis was detected after 2 h incubation. NCR247 also induced cell degradation which was confirmed by the appearing blebs and intracellular material around the bacteria after 5 min of treatment.

Extensive membrane damage is a key factor in the inactivation of bacteria by AMPs. Experimental investigations supported by computational analysis confidently classified and demonstrated the antimicrobial nature of two *M. truncatula* NCR peptides. The highly cationic NCR247 and NCR335 plant peptides efficiently eliminated Gram-negative and Gram-positive foodborne pathogenic bacteria. The peptides were shown to disrupt the physical structure of the bacterial cell by increasing membrane permeability through pore formation.

## Author Contributions

GM and AF designed and executed the experiments as well as composed the manuscript. AK and EK added useful recommendations and edited the manuscript.

## Conflict of Interest Statement

The authors declare that the research was conducted in the absence of any commercial or financial relationships that could be construed as a potential conflict of interest.
